# Staged PFCL-assisted ILM free flap transplantation for refractory macular holes: resolving the mechanical paradox of graft instability

**DOI:** 10.1186/s40942-026-00909-z

**Published:** 2026-07-29

**Authors:** Hideyuki Nakano, Hiroko Terashima, Taku Sato, Kazuhito Yoneda, Jiro Kogo, Kenji Murakami

**Affiliations:** 1 Department of Ophthalmology, Niigata City General Hospital, 463-7 shumoku, Chu-o-ku, Niigata City, Niigata 950-1197 Japan; 2 Department of Ophthalmology, Niigata University Graduate School of Medical and Dental Science, Niigata, Japan; 3 Takasaki Sato Eye Clinic, Minamiorui, Takasaki, Gunma Japan; 4 Subarukai Eye Center, Higashi-Ohmi City, Shiga Japan

**Keywords:** Internal limiting membrane transplantation, Dual-interface surgical framework, Refractory macular holes, Pars plana vitrectomy, Perfluorocarbon liquid

## Abstract

**Introduction:**

Primary pars plana vitrectomy with internal limiting membrane (ILM) peeling closes over 90% of idiopathic full-thickness macular holes (MHs), and revision surgery is challenging owing to limited remaining ILM, especially in myopic or extensively peeled eyes. The present study aimed to propose a staged perfluorocarbon liquid (PFCL)-assisted dual-interface stabilization strategy for autologous ILM free flap transplantation in refractory MHs and provide its biomechanical rationale along with prospective pilot clinical validation.

**Methods:**

This prospective, multi-center interventional case series included five consecutive eyes with refractory full-thickness MHs (mean age 68.3 ± 8.8 years; mean minimum linear diameter 449.2 ± 196.5 μm) following prior pars plana vitrectomy with ILM peeling and gas tamponade. The framework consisted of (1) placement of an ophthalmic viscoelastic device (OVD) plug within the MH, (2) limited PFCL stabilization, (3) ILM flap preparation outside the PFCL meniscus to preserve membrane mobility, and (4) secondary PFCL expansion before fluid–air exchange. Anatomical closure and best-corrected visual acuity (BCVA) were evaluated during at least 3 months of follow-up.

**Results:**

Complete (type 1) MH closure was achieved in all eyes (100%) without intraoperative flap displacement or subretinal PFCL migration. The mean BCVA improved from 0.81 ± 0.34 logMAR preoperatively to 0.41 ± 0.19 logMAR at final follow-up (mean gain 0.40 logMAR). Postoperative OCT confirmed stable ILM graft positioning without secondary contraction or migration.

**Conclusions:**

By separating flap mobility from fixation in a staged manner, PFCL application may resolve the mechanical dilemma underlying ILM transplantation. Prospective pilot results supported the feasibility of the technique and showed consistent anatomical closure in refractory MHs.

**Supplementary Information:**

The online version contains supplementary material available at https://doi.org/10.1186/s40942-026-00909-z.

## Background

Primary pars plana vitrectomy (PPV) with internal limiting membrane (ILM) peeling achieves anatomical closure rates exceeding 90% in idiopathic full-thickness macular holes (MHs) [1]. Nevertheless, persistent or reopened MHs remain clinically challenging, particularly in eyes with large defects, chronic disease duration, high axial myopia, or extensive prior ILM removal [2–4]. In such cases, the biological and mechanical conditions required for successful closure are substantially altered, and conventional edge approximation strategies may be insufficient.

Revision surgery is technically demanding because the amount of residual ILM available for manipulation is often limited following previous peeling, especially in highly myopic eyes or in cases with broad primary peeling [2, 3]. Several advanced techniques have been proposed, including the inverted ILM flap technique [5], floating flap technique for myopic MHs [6], and autologous ILM free flap transplantation [7–10]. The latter provides a biological scaffold that may facilitate Müller cell-mediated gliosis and tissue bridging across the foveal defect [7, 8].

Despite its conceptual advantages, ILM free flap transplantation remains technically challenging because intraoperative graft instability, particularly during fluid–air exchange (FAX), may result in flap displacement and compromise surgical success [11, 12]. Perfluorocarbon liquid (PFCL)-assisted techniques have been introduced to improve graft stabilization during these critical surgical steps [13, 14]. However, maintaining the flap under a continuous PFCL column throughout the procedure may reduce membrane mobility during flap preparation, potentially making delicate manipulation more difficult owing to PFCL-induced flattening forces [13–16]. Consequently, although PFCL improves graft stability, balancing flap maneuverability during preparation with secure fixation during FAX remains a practical surgical challenge.

To our knowledge, no previous study has described a staged PFCL-assisted strategy that deliberately separates flap preparation from graft fixation to optimize both membrane maneuverability and intraoperative stability during ILM free flap transplantation. We therefore developed a staged PFCL-assisted dual-interface technique that temporally separates these steps. In this approach, the flap is prepared before PFCL expansion to preserve membrane mobility, followed by PFCL-assisted stabilization before FAX. In addition, placement of a dispersive ophthalmic viscoelastic device (OVD) plug within the MH may provide a protective interface over the retinal pigment epithelium (RPE) while facilitating controlled graft positioning. We hypothesized that this staged strategy would improve the technical reproducibility and ease of ILM free flap transplantation while maintaining graft stability during FAX. The proposed biomechanical rationale should be regarded as a conceptual hypothesis underlying the surgical design rather than a proven mechanism. In this prospective pilot study, we evaluated the anatomical and functional outcomes of this technique and assessed the clinical feasibility of the proposed staged PFCL-assisted dual-interface strategy in eyes with refractory MHs.

## Methods

### Study design and ethics

This prospective multi-center interventional case series adhered to the tenets of the Declaration of Helsinki. Written informed consent was obtained from all patients prior to surgery. The study protocol was approved by the Institutional Review Board of Niigata City General Hospital, Niigata, Japan (approval number: 25–066), and participating centers. The study was performed in accordance with the ethical standards as laid down in the 1964 Declaration of Helsinki and its later amendments or comparable ethical standards.

### Patient selection

Patients with refractory full-thickness MHs were prospectively enrolled. Refractory MH was defined as a persistent or reopened MH with exposed RPE following PPV with ILM peeling and gas tamponade.

Five eyes of five consecutive patients who underwent autologous ILM free flap transplantation were included. All eyes were pseudophakic prior to revision surgery, and no combined cataract surgery was conducted during the study procedure. All MHs were idiopathic, including one case associated with high myopia. Exclusion criteria included ocular comorbidities that could affect visual acuity, such as diabetic retinopathy, age-related macular degeneration, choroidal neovascularization, advanced glaucoma, and significant media opacity.

### Surgical technique

All procedures were conducted under local anesthesia by two experienced vitreoretinal surgeons (H.N. and T.S.). Standard 25- or 27-gauge three- or four-port PPVs were conducted. Adequate core and peripheral vitrectomies were confirmed either during the current procedure or before surgery.

#### Step 1: OVD plug placement

**Fig. 1:**
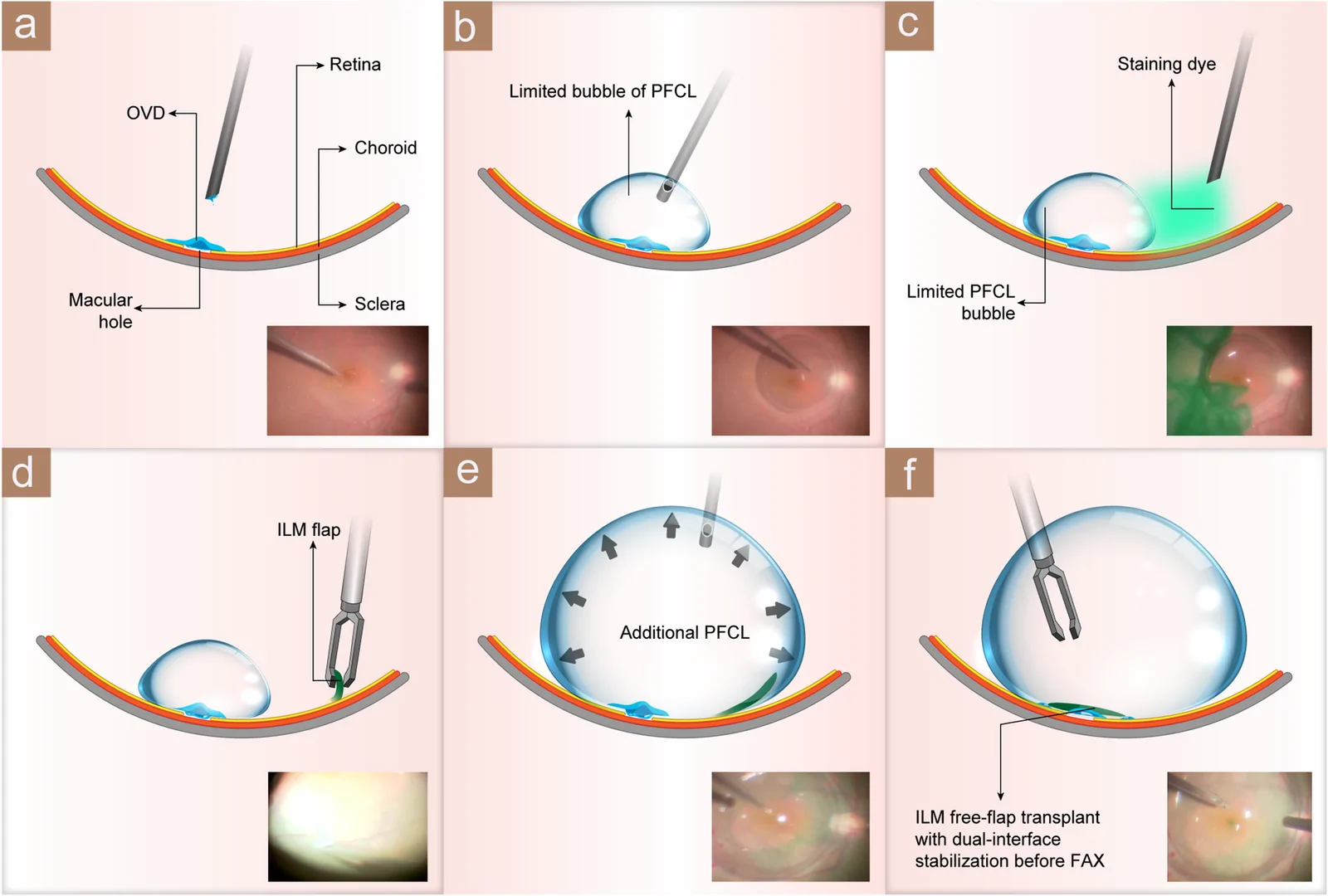
Stepwise staged perfluorocarbon liquid (PFCL)-assisted internal limiting membrane (ILM) free flap transplantation technique. The upper panels show schematic illustrations, and the lower panels present corresponding intraoperative photographs demonstrating the structured surgical sequence. (**a**) A small volume of dispersive ophthalmic viscoelastic device is gently instilled into the macular hole (MH) to prevent intra-hole dye exposure and to protect the retinal pigment epithelium. (**b**) A limited bubble of PFCL is injected to stabilize the posterior pole while preserving a PFCL-free zone for subsequent flap preparation. (**c**) ILM staining is conducted outside the PFCL meniscus to minimize dye migration into the MH. (**d**) An ILM free flap is created in the PFCL-free zone to avoid PFCL-induced flattening and to preserve membrane elasticity and maneuverability. (**e**) After transposition of the flap over the MH, additional PFCL is carefully introduced to fully cover the macula and attain stable graft fixation prior to fluid–air exchange. (**f**) The ILM free flap is secured over the MH under PFCL, providing stable support during completion of fluid–air exchange. FAX, fluid–air exchange; OVD, ophthalmic viscoelastic device

A small volume (0.05–0.1 mL) of dispersive ophthalmic viscoelastic device (Viscoat; Alcon Laboratories, Fort Worth, TX, USA) was gently instilled into the MH to prevent intra-hole dye ingress and to provide a protective interface over the RPE (Fig. 1a). As dispersive OVD becomes trapped beneath the PFCL and cannot be aspirated afterward, only a minimal amount is intentionally employed to serve as a localized protective barrier.

#### Step 2: First PFCL stabilization

A limited PFCL bubble (Perfluoron; Alcon Laboratories) was injected to stabilize the posterior pole, while deliberately preserving a PFCL-free zone over the intended ILM harvest area (Fig. 1b). ILM staining was performed with either 0.025% brilliant blue G or 0.125% indocyanine green, depending on institutional availability. Staining was performed outside the PFCL meniscus to minimize fluid turbulence and prevent dye migration into the MH (Fig. 1c). The choice of Brilliant Blue G or indocyanine green was based on the surgeon’s routine clinical practice and intraoperative preference rather than predefined selection criteria.

#### Step 3: Free flap preparation

From the remaining ILM area, the edge of a sufficiently large ILM free flap is created within the PFCL-free zone. Flap preparation outside the PFCL boundary preserved the membrane elasticity and prevented PFCL-induced flattening or contraction (Fig. 1d). The flap was carefully mobilized toward the MH while maintaining its structural integrity and adequate surface area for subsequent coverage.

#### Step 4: Second PFCL stabilization and flap positioning

Additional PFCL was gradually injected to completely cover the macular region and stabilize the prepared flap (Fig. 1e). Under PFCL, the ILM free flap was gently positioned over the MH before fluid–air exchange (Fig. 1f), thereby establishing gravitational stabilization during the critical transition phase. This staged configuration temporally separates the mobility phase of flap preparation from the fixation phase required during fluid–air exchange. The transition from flap mobility to PFCL-mediated stabilization is demonstrated in an additional movie file (see Additional file 1: Video S1).

#### Fluid–air exchange and tamponade

Fluid–air exchange was conducted under continuous visualization while maintaining the aspiration port nasal to the optic disc to minimize macular turbulence and reduce the risk of flap displacement. A sulfur hexafluoride (SF₆) gas tamponade was used in all cases. Postoperatively, the patients were instructed to maintain a prone position and avoid supine positioning until anatomical macular hole closure was confirmed on OCT.

### Outcome assessment

All patients underwent a comprehensive ophthalmic evaluation, including best-corrected visual acuity (BCVA), intraocular pressure, slit-lamp biomicroscopy, fundus photography, axial length measurement, and swept-source optical coherence tomography (SS-OCT). The BCVA recorded in decimal notation was converted to the logarithm of the minimal angle of resolution (logMAR) units. Postoperative closure status was assessed using SS-OCT, and MH size (minimum and base diameters) measurements were performed using the caliper tool in the inbuilt software. The MH closure was classified as follows:

- Type 1 (complete closure): complete retinal continuity without exposed RPE.
- Type 2 (incomplete closure): flattening of the hole edges with a persistently exposed RPE.

## Results

### Patient characteristics

Five eyes of five patients (mean age: 68.3 ± 8.8 years; 2 males and 3 females) with refractory full-thickness MHs were included. The cohort comprised three idiopathic MHs and one highly myopic MH (case 3; axial length, 31.03 mm). The mean minimum linear diameter (MLD) was 449.2 ± 196.5 μm (range: 179–702 μm), and the mean base diameter was 1000 ± 593.6 μm (range: 663–1978 μm). **A**ll eyes underwent revision surgery after a single previous pars plana vitrectomy with ILM peeling and gas tamponade. The interval between the primary surgery and revision procedure ranged from 8 to 21 days (mean, 13.8 days). During the primary surgery, in all eyes, the ILM had been peeled circumferentially around the MH over an area of approximately two disc diameters centered on the macular hole. Revision surgery was performed after postoperative OCT confirmed a persistent MH without evidence of anatomical closure. All eyes were pseudophakic before the revision procedure and remained pseudophakic at the final follow-up; no eyes underwent combined cataract surgery (Table 1).

**Table 1:** Patient characteristics and surgical outcomes

				MH diameter (µm)			BCVA (Decimal)			
Case No.	Age	Sex	Primary disease	Minimum	Base	Tamponade	Preoperative	Postoperative at final visit	Postoperative MH status	Final lens status
1	72	M	Idiopathic	358	663	SF6	0.4	0.6	Closed	Pseudophakic
2	55	M	Idiopathic	702	1978	SF6	0.15	0.2	Closed	Pseudophakic
3	73	F	Highly myopic	463	928	SF6	0.3	0.4	Closed	Pseudophakic
4	73	F	Idiopathic	544	1009	SF6	0.08	0.7	Closed	Pseudophakic
5	61	F	Idiopathic	179	422	SF6	0.08	0.3	Closed	Pseudophakic

MH: Macular hole; BCVA: Best-corrected visual acuity; SF6: Sulfur hexafluoride; M: Male; F: Female

### Intraoperative findings

All the procedures were successfully completed using a stepwise PFCL-assisted technique. The external ILM free flap preparation outside the PFCL meniscus facilitated the controlled creation of a sufficiently large and mobile ILM graft in all cases, despite the limited residual ILM from prior surgery. No intraoperative complications occurred, including flap displacement during fluid–air exchange, subretinal PFCL migration, iatrogenic retinal tears, or intraoperative bleeding. Staged PFCL application allowed stable positioning of the ILM flap prior to and during fluid–air exchange in all eyes.

### Anatomical outcomes

**Fig. 2:**
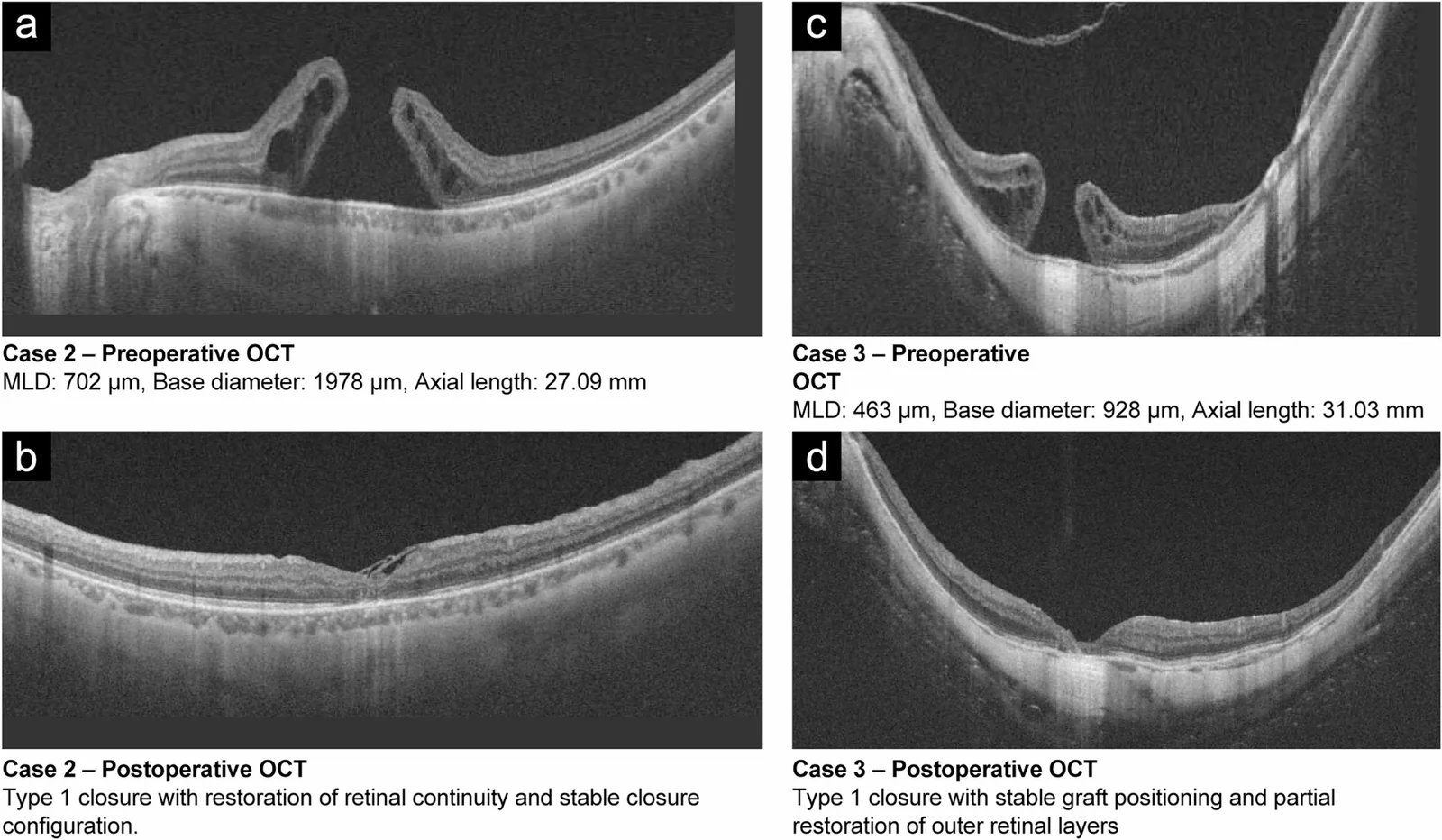
OCT images of representative refractory macular holes treated with staged PFCL-assisted ILM free flap transplantation. (**a**, **b**) Case 2. (**a**) Preoperative optical coherence tomography (OCT) demonstrates a large full-thickness macular hole with a minimum linear diameter (MLD) of 702 μm and a base diameter of 1978 μm (axial length 27.09 mm). (**b**) Postoperative OCT at final follow-up shows complete (type 1) macular hole closure with restoration of retinal continuity across the foveal defect. (**c**, **d**) Case 3. (**c**) Preoperative OCT shows a refractory macular hole in a highly myopic eye (axial length 31.03 mm) with an MLD of 463 μm and a base diameter of 928 μm. (**d**) Postoperative OCT demonstrates type 1 closure with stable graft positioning and partial restoration of outer retinal layers

Complete MH closure was achieved in all five eyes (100%; 95% exact binomial CI, 48–100%). According to the OCT-based classification, all eyes demonstrated type 1 (complete) closure, which is defined as complete retinal continuity without exposed RPE. Postoperative SS-OCT confirmed stable ILM graft coverage of the foveal defect, without displacement, contraction, or graft migration throughout follow-up. No postoperative MH reopening occurred during the observation period. Partial restoration of the outer retinal layers, including the external limiting membrane (ELM) and ellipsoid zone (EZ), was observed in all eyes at the final visit (Fig. 2). The mean follow-up duration was 4.0 ± 1.7 months (range, 2–9 months).

### Functional outcomes

Mean BCVA improved from 0.81 ± 0.34 logMAR preoperatively to 0.41 ± 0.19 logMAR at final follow-up, corresponding to a mean gain of 0.40 logMAR. The BCVA improved in all five eyes. Given the exploratory design and limited sample size of this pilot study, outcomes were reported descriptively (Table 1).

### Safety outcomes

No dye-related retinal toxicity or PFCL-related adverse events, including the retention of subfoveal PFCL, were noted. There were no postoperative complications, such as endophthalmitis, sustained intraocular pressure elevation, or the need for additional surgical intervention. No eyes required reoperation during the follow-up period.

## Discussion

Refractory or recurrent full-thickness MHs continue to pose a significant surgical challenge, particularly in eyes with limited residual ILM after prior vitrectomy and ILM peeling [2, 3]. Although primary MH surgery achieves high closure rates [1], persistence or reopening is more common in large, chronic, or highly myopic holes [2–4], where both the biological substrate and the mechanical environment required for closure are compromised. In such cases, revision strategies must address not only the absence of residual ILM but also the technical challenges associated with stable graft placement.

Autologous ILM free flap transplantation was developed to provide a biological scaffold capable of supporting Müller cell-mediated gliosis and tissue bridging across the defect [8–10]. Although this approach has improved closure rates in complex MHs, its success depends heavily on intraoperative graft stability. Flap displacement, folding, or contraction, particularly during FAX, has been recognized as a major cause of surgical failure [11, 12]. These observations suggest that ILM transplantation requires a balance between sufficient membrane mobility during flap preparation and stable graft fixation during FAX. Optimizing both conditions may improve the technical reproducibility of this procedure.

To our knowledge, this is the first study to describe a staged PFCL-assisted strategy that deliberately separates flap preparation from graft fixation to optimize both membrane maneuverability during harvesting and graft stabilization during FAX. Rather than replacing existing PFCL-assisted techniques, this approach was developed as a practical refinement to address these competing technical requirements.

In this pilot series, temporally separating flap preparation and fixation was technically feasible and was consistently accompanied by stable intraoperative graft behavior. Preparing the flap outside the PFCL meniscus preserved membrane mobility and allowed controlled harvesting even in eyes with minimal residual ILM. This consideration may be particularly relevant in highly myopic eyes, where the retina is thinned and the ILM is often fragile [17]. The same technical challenge may also arise during revision surgery after extensive ILM peeling, where insufficient residual ILM may limit the feasibility of a conventional pedicled flap and necessitate free ILM transplantation. Under a continuous PFCL column, hydrostatic pressure and surface tension may flatten the ILM against the retinal surface, potentially reducing maneuverability and increasing the risk of flap fragmentation [13–16]. Avoiding PFCL during the preparation phase may therefore preserve tactile feedback and membrane compliance, facilitating precise flap creation. Whether this staged workflow provides superior intraoperative control compared with existing PFCL-assisted techniques cannot be determined from the present study and requires future comparative studies.

Once the flap had been prepared and mobilized, secondary PFCL expansion provided gravitational stabilization during the transition to FAX. This staged strategy differs from previously described PFCL-assisted techniques, such as that of Rossi et al., in which flap preparation and stabilization are performed under continuous PFCL [15]. While continuous PFCL effectively stabilizes the graft, our approach was designed to explore an alternative workflow that separately optimizes flap preparation and fixation. Although the present study was not designed to compare surgical techniques directly, the favorable intraoperative observations suggest that this staged strategy is technically feasible and may represent a useful option in selected complex cases.

For this technique, we propose the concept of dual-interface stabilization. Placement of a dispersive OVD plug within the MH establishes a viscous lower boundary that protects the RPE and provides gentle counter-support beneath the graft [18]. Above the graft, PFCL provides a high-density upper boundary that exerts gravitational stabilization. Together, these interfaces may confine the graft between two stabilizing layers, potentially reducing shear stress during FAX and limiting horizontal displacement forces. Although the stabilizing effect of PFCL is well recognized [13], the biomechanical interpretation presented here should be regarded as a conceptual hypothesis rather than a proven mechanism. Future experimental and computational studies will be necessary to validate this proposed framework. Although Brilliant Blue G is generally considered safer than indocyanine green [19], minimizing direct dye exposure to the RPE remains desirable. The OVD layer may therefore provide both mechanical stabilization and additional protection during staining and flap positioning [20].

The anatomical outcomes in this pilot series were encouraging. Complete type 1 closure was achieved in all eyes, with no intraoperative flap displacement or subretinal PFCL migration. Functional outcomes were also favorable, with a mean gain of 0.40 logMAR and partial restoration of the ellipsoid zone in all eyes, consistent with previous reports of ILM transplantation [7–9]. Visual recovery after revision MH surgery is generally less favorable than after primary surgery [2–4]. The consistent intraoperative graft behavior observed across all cases supports the technical feasibility of the proposed workflow. However, because this study included only a small number of eyes and lacked a comparison group, it cannot determine whether the observed stability or clinical outcomes are superior to those achieved with existing PFCL-assisted techniques.

These findings should be interpreted within the broader clinical context. Patients requiring revision MH surgery represent a relatively uncommon and highly selected population, making large prospective comparative studies difficult to conduct. In this setting, carefully characterized pilot series can provide valuable technical insights and generate hypotheses for future investigation. Beyond the clinical outcomes, the present study introduces a practical surgical workflow together with a conceptual biomechanical framework that may facilitate further refinement of ILM transplantation techniques. Similar requirements for controlled graft manipulation followed by stable fixation arise in other retinal graft-based procedures, suggesting that the underlying principles described here may have broader applicability.

This study has several limitations. The sample size was small, limiting generalizability and precluding direct comparison with alternative surgical techniques. The follow-up period was relatively short, and longer observation is needed to determine the durability of anatomical closure and the risk of late reopening. In addition, the proposed biomechanical rationale remains hypothetical and was not directly tested in this clinical study. Quantitative biomechanical modeling and experimental validation would help clarify the physical mechanisms underlying the observed intraoperative behavior. Because this was a small, non-comparative pilot study, the present findings should not be interpreted as evidence of superiority over existing PFCL-assisted techniques. Rather, they support the technical feasibility of the proposed surgical workflow and provide a basis for future comparative studies. Finally, all surgeries were performed by experienced vitreoretinal surgeons, and the reproducibility of this technique across surgeons with varying levels of experience warrants further evaluation.

Despite these limitations, the staged PFCL-assisted dual-interface strategy represents a concept-driven technical refinement of ILM free flap transplantation. By intentionally separating flap preparation from graft fixation, the technique seeks to balance membrane maneuverability with intraoperative stability while maintaining a simple and reproducible surgical workflow. Although the proposed biomechanical explanation remains hypothetical and the clinical advantages over existing techniques have yet to be established, the favorable pilot findings support the feasibility of this concept and justify further comparative evaluation. Future comparative studies with larger cohorts and longer follow-up will be required to determine whether this staged strategy provides measurable clinical advantages over established PFCL-assisted techniques and whether the proposed dual-interface framework is applicable to other graft-based vitreoretinal procedures.

## Conclusions

Staged PFCL-assisted ILM free flap transplantation represents a concept-driven refinement of ILM transplantation that separates flap preparation from PFCL-mediated fixation to balance membrane maneuverability with intraoperative graft stabilization. In this pilot series, the technique was technically feasible and achieved complete anatomical closure in all refractory MHs. Although the proposed biomechanical framework remains hypothetical, these findings support further comparative evaluation to determine the clinical value, long-term efficacy, and broader applicability of this staged dual-interface strategy.

## Supplementary Information

Below is the link to the electronic supplementary material.

Supplementary Material 1: Video S1. Staged PFCL-assisted ILM free flap transplantation: visualization of mobility-to-stabilization transition.

## Data Availability

The datasets used and/or analyzed during the current study are available from the corresponding author on reasonable request.

## Abbreviations

BCVA: Best-corrected visual acuity
FAX: Fluid–air exchange
ILM: Internal limiting membrane
MH: Macular hole
MLD: Minimum linear diameter
PFCL: Perfluorocarbon liquid
PPV: Pars plana vitrectomy
OVD: Ophthalmic viscoelastic device
RPE: Retinal pigment epithelium
SS-OCT: Swept-source optical coherence tomography
